# Spontaneous and Evoked Activity from Murine Ventral Horn Cultures on Microelectrode Arrays

**DOI:** 10.3389/fncel.2017.00304

**Published:** 2017-09-29

**Authors:** Bryan J. Black, Rahul Atmaramani, Joseph J. Pancrazio

**Affiliations:** Neuronal Networks and Interfaces Laboratory, Department of Bioengineering, The University of Texas at Dallas, Richardson, TX, United States

**Keywords:** ventral horn, motor neuron, microelectrode array, *in vitro* models, neural activity recording, neural activity modulation, electrical stimulation, BoNT/A

## Abstract

Motor neurons are the site of action for several neurological disorders and paralytic toxins, with cell bodies located in the ventral horn (VH) of the spinal cord along with interneurons and support cells. Microelectrode arrays (MEAs) have emerged as a high content assay platform for mechanistic studies and drug discovery. Here, we explored the spontaneous and evoked electrical activity of VH cultures derived from embryonic mouse spinal cord on multi-well plates of MEAs. Primary VH cultures from embryonic day 15–16 mice were characterized by expression of choline acetyltransferase (ChAT) by immunocytochemistry. Well resolved, all-or-nothing spontaneous spikes with profiles consistent with extracellular action potentials were observed after 3 days *in vitro*, persisting with consistent firing rates until at least day *in vitro* 19. The majority of the spontaneous activity consisted of tonic firing interspersed with coordinated bursting across the network. After 5 days *in vitro*, spike activity was readily evoked by voltage pulses where a minimum amplitude and duration required for excitation was 300 mV and 100 μs/phase, respectively. We characterized the sensitivity of spontaneous and evoked activity to a host of pharmacological agents including AP5, CNQX, strychnine, ω-agatoxin IVA, and botulinum neurotoxin serotype A (BoNT/A). These experiments revealed sensitivity of the cultured VH to both agonist and antagonist compounds in a manner consistent with mature tissue derived from slices. In the case of BoNT/A, we also demonstrated intoxication persistence over an 18-day period, followed by partial intoxication recovery induced by N- and P/Q-type calcium channel agonist GV-58. In total, our findings suggest that VH cultures on multi-well MEA plates may represent a moderate throughput, high content assay for performing mechanistic studies and for screening potential therapeutics pertaining to paralytic toxins and neurological disorders.

## Introduction

Spinal motor neurons (MNs) constitute the final common neural pathway for the control of movement. With cell bodies located in the ventral horn (VH) of the spinal cord, alpha MNs innervate muscle fibers to enable voluntary movement. MNs play an important role in neurological disorders and the action of paralytic toxins. For example, spinal muscular atrophy and amyotrophic lateral sclerosis are manifested by degeneration of MNs. Tetanus toxin and the family of botulinum neurotoxins act on MNs at the synapse to suppress neurotransmitter release, producing paralysis during poisoning (Burgen et al., 1948; Parsons et al., 1966).

Given their importance, numerous screening and mechanistic studies have made use of primary MNs (Kuo et al., 2004), tumor-derived MN-like cells (Maier et al., 2013), or stem cell-derived MNs (Sances et al., 2016) in assays often relying on cytotoxicity, neurite extension, or DNA damage as an endpoint. It has been increasingly recognized that high content screening approaches that incorporate physiologically relevant metrics have important roles in next generation assays. For electrically active cells, the expression of all-or-nothing action potentials is critical to both physiology and function. Substrate integrated microelectrode arrays (MEAs) permit the long-term non-invasive recording of spontaneous and evoked activity from cultures of electrically active cells. In this paper, we characterize the expression, stability, and pharmacology of cultures of embryonically derived murine VH on multi-well plates of MEAs. We show that by 3 days *in vitro* (DIV), spontaneous extracellular action potentials or spikes emerge, reaching a peak mean spike rate at 18 DIV. After day 5, electrically evoked activity, triggered by biphasic voltage pulses of at least 300 mV and 100 μs/phase, elicit brief bursts of spikes. Exposure to AP5/CNQX, atropine sulfate, bicuculline, strychnine, ω-agatoxin IVA, and botulinum neurotoxin serotype A (BoNT/A) either increased or decreased spontaneous activity consistent with *in vivo* pharmacology. In total, our findings suggest that VH cultures on multi-well plates of MEAs may enable a stable high content platform for screening potential therapeutics and mechanistic studies pertaining to paralytic toxins and neurological disorders.

## Materials and Methods

### Reagents

Botulinum neurotoxin serotype A from *Clostridium botulinum* was purchased from List Biological Laboratories, Inc. (Campbell, CA, United States). Supplemented Dulbecco’s Modified Eagle’s Medium (SDMEM) consists of DMEM + Glutamax (Cat No. 10569010, Sigma–Aldrich) with 2% B-27, 8 μg/ml ascorbic acid, and 1% penicillin streptomycin. SDMEM 5/5 consists of SDMEM plus 5% horse serum and 5% fetal bovine serum. Pharmacological agents used in this study include strychnine (Cat No. AC158950250, ACROS Organics), 1(S), 9(R)-(-)-bicuculline methchloride (Cat No. B7686, Sigma), GV-58 (Cat No. SML1551, Sigma), α-bungarotoxin–tetramethylrhodamine from *Bungarus multicinctus* (Cat No. T0195, Sigma), atropine sulfate (VEDCO), D-AP5 (Cat No. ab12003, Abcam), ω-agatoxin IVA (Cat No. A6719, Millipore Sigma, Germany), and CNQX (Cat No. C127, Millipore Sigma, Germany).

### Immunocytochemistry, Imaging, and Quantification

Ventral horn cultures, aged DIV 18, were fixed with 4% paraformaldehyde solution for 10 min and then washed three times with ice-cold phosphate buffered saline (PBS). Cells were permeabilized with 0.25% Triton X100 for 30 min, blocked with 10% normal goat serum (NGS) in PBS for 3 h, and incubated with primary antibodies diluted in NGS for 2 h at room temperature. Primary antibodies used were rabbit anti-ChAT (Alexa Fluor 647) (ab178850, Abcam, 1:500), and chicken anti-Neun (ABN91, EMD Millipore, 1:500). Cells were then incubated with fluorochrome conjugated secondary antibody goat anti-chicken IgY (Alexa fluor 488) (1:200) for 1 h at room temperature. Labeled cells were imaged at 20× using an inverted microscope (Nikon, Japan) and epifluorescent light sources (Lumencor, United States).

Automated cell counts were performed using boutique macros in ImageJ v.1.6 (NIH, United States). Briefly, a two-pixel Gaussian blur was applied to each image channel, followed by automated detection of local intensity maxima. User input determined the threshold noise tolerance for maxima detection. Cell counts were further analyzed and plotted using OriginPro software (OriginLab Corporation, Northampton, MA, United States).

### Primary Neuronal Culture

Ventral regions of spinal cord were dissected from embryonic day 15–16 (E15–16) mice. Anesthetized timed pregnant female mice (ICR-CD1, Envigo, United Kingdom) were euthanized by cervical dislocation or decapitation in accordance with UTD Institutional Animal Care and Use Committee (IACUC). Embryos were removed via caesarian section and washed once in ice-cold sterile HBSS. Individual embryos were removed from their uterine sack, decapitated, and placed in fresh ice-cold sterile HBSS. Spinal cords were extracted as previously described (Zhang and Robinson, 2009) and ventral regions isolated. Ventral spinal cord regions were then minced and placed in enzymatic dissociation solution (0.025% trypsin-EDTA, 200 U/ml DNAse) at 37°C for approximately 30 min. Mechanical dissociation was then carried about by trituration using fire-polished glass Pasteur pipettes until tissue was homogeneously suspended with no visible sections/aggregates.

Cells were collected by centrifugation at 300 × *g* for 7 min and the resulting cell pellet resuspended in fresh SDMEM 5/5. The 10 μl of cell suspension was mixed 1:1 with Trypan Blue, and viable cell concentration was quantified using a hemocytometer. Further dilution was carried out to bring viable cell concentration to 90,000 cells per 5 μl. Droplets of 5 μl were then added to the center of each pre-treated MEA and stored in the cell culture incubator for 30 min to allow cell adhesion. Wells were then carefully flooded with 200 μl SDMEM 5/5 and replaced in the incubator. Cell cultures underwent 50% medium exchanges every even day *in vitro* following seeding using SDMEM without serum to prevent overgrowth of non-neuronal cells.

### Multi-well Microelectrode Array Plate Preparation

Pre-sterilized, opaque 48-well MEA plates were purchased from Axion Biosystems (Cat No. – M768-KAP-48; Georgia, United States). The 10 μl droplets of 50 μg/ml poly-D-lysine were added to the center of each MEA and allowed to incubate overnight at 37°C. The following morning, PDL was removed and the wells were washed twice with sterile DDI water and allowed to dry within the laminar flow cabinet. Once dried, 5 μl droplets of 20 μg/ml laminin were added to the center of each MEA and incubated for 1 h at 37°C. Immediately prior to cell seeding, laminin was removed but the surface was not allowed to dry completely.

### Extracellular Recordings and Stimulation

Extracellular recordings were carried out using Axion’s Maestro multi-well 768 electrode recording platform in combination with Axion 48-well MEA plates. Each well houses a 4×4 16 channel electrode array with four additional reference electrodes. Simultaneous extracellular voltage recordings were collected from up to 768 channels at a sampling rate of 12.5 kHz per channel. Continuous data were filtered by a 1-pole Butterworth bandpass filter (300–5000 Hz). Individual spikes were detected by filtered continuous data crossing of a ±6σ adaptive threshold based on continuous 1 s snapshots of each channel’s RMS noise. Unless otherwise stated, all analysis considers only ‘active’ channels, defined as channels exhibiting ≥5 spikes/min. Single-electrode bursts were defined as at least five consecutive spikes with interspike intervals less than 100 ms. Network bursts were defined as at least 10 consecutive spikes across multiple electrodes with interspike intervals of less than 100 ms and a minimum of 50% electrodes participating (Sanes and Lichtman, 1999). Quantification of network synchrony was carried out by calculating area under the normalized cross-correlogram (Luhmann et al., 2016) as well as the synchrony index (Paiva et al., 2010) for each well using Axion’s NeuralMetric Tool software. Further analysis (mean firing rate, bursting rate, etc.) was carried out using a combination of Axion’s Axis Metric program and boutique Matlab script. Graphing and statistical tests were carried out using OriginPro software (OriginLab Corporation, Northampton, MA, United States). During preliminary experiments, we found that the occurrence of multiple characteristic waveforms (or single units) being recorded by a single electrode was rare (<2%). Therefore, no single-unit discrimination was carried out prior to quantification of mean firing rate, etc.

Simultaneous electrical stimulation and recording was performed using hardware and software solutions from Axion Biosystems Inc. Cathodic-leading biphasic square pulses were used for voltage stimulation at amplitudes ranging from 100 to 1200 mV (maximum allowed current = 250 μA) and pulse durations from 100 to 250 μs/phase.

### Pharmacological Exposure

Unless otherwise stated, all pharmacological compounds were introduced simultaneously at final working concentrations via 100% medium exchange. Cultures were allowed to acclimate for 10 min and then recorded for 30 min. In the case of BoNT/A, cultures were exposed to working concentrations of 25, 50, or 100 ng/ml and monitored continuously for 14 h. Twenty-four hours following introduction, medium containing BoNT/A was removed, all wells were washed once, and then replenished with fresh medium. BoNT-treated wells and negative controls were then recorded every alternate day for 18 days following initial BoNT treatment. Handling and disposal of toxins (botulinum toxin serotype A) was carried out in accordance with UTD Institutional Biosafety and Chemical Safety Committee (IBCC) protocols.

### Statistical Analysis

In the case of pharmacological treatments, ‘Normalized Mean Firing Rate’ and ‘Normalized Mean Bursting Rate’ was calculated as the difference-over-sum with respect to an associated baseline recording ((Treatment – Baseline)/(Treatment + Baseline)). This normalizes the relative change of the treatment to values between -1 and 1. In the case of BoNT/A, data are reported as ‘Weighted Mean Firing Rate,’ defined as the spike rate per well multiplied by the number of active electrodes in the associated well. All statistical analysis was performed in either MATLAB (Mathworks Inc.) or OriginPro 2016 (OriginLab Corp., United States). The comparison of each group to the baseline level was done using two-sample *t*-test. To compare between group effects, repeated measures analysis of variance (ANOVA) were utilized. In all case, *P* < 0.05 was considered significant. Data are expressed as mean ± standard error of the mean (SEM).

## Results

### Immunocytochemistry

**FIGURE 1:**
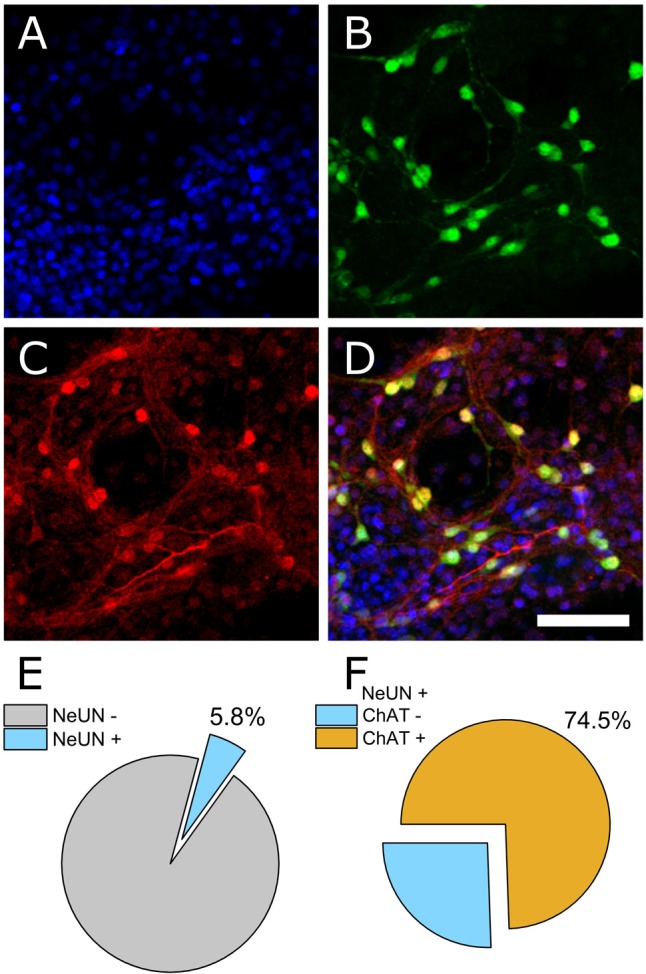
Immunocytochemical staining to determine neuronal and non-neuronal culture populations. **(A)** DAPI staining indicates all cell nuclei. **(B)** NeUN indicates neuronal nuclei. **(C)** Choline acetyltransferase (ChAT). **(D)** Merged image. **(E)** Quantification of co-expression of DAPI and NeUN versus DAPI+ counts (percentage of neuronal cells). **(F)** Of the neuronal population, 74.5% were ChAT positive.

The ventral spinal cord is comprised of neuronal as well as non-neuronal cellular sub-types. To determine the ratio of neuronal to non-neuronal as well as the ratio of MN to inter-neuron populations within our preparation, we performed immunocytochemistry on PFA-fixed cultures (DIV 7). 4′,6-diamidino-2-phenylindole (DAPI) was used to quantify total cell number within an imaged field of view and the neuronal nuclear antigen NeUN was used to discriminate neuronal from non-neuronal nuclei. Choline acetyltransferase (ChAT) was used to further discriminate MNs from other neuronal cell types. **Figures 1A–D** show representative DAPI (blue), NeUN (green), and ChAT (red) antibody staining. Automated counting of DAPI versus NeUN-positive nuclei indicated that 94.2% of cells present in culture on DIV 7 were non-neuronal (**Figure 1E**, *N* = 4 cultures, 16 ROIs). Of the neuronal population, however, 74.5% were found to be ChAT positive (**Figure 1F**). These data indicate that our dissection and culturing protocols led to successful culturing of MNs isolated from ventral spinal cord and suggest that the resulting extracellular recordings should reflect contributions from MNs as well as other ventral spinal neurons; most likely inhibitory interneurons.

### Extracellular Recordings from VH Cultures

**FIGURE 2:**
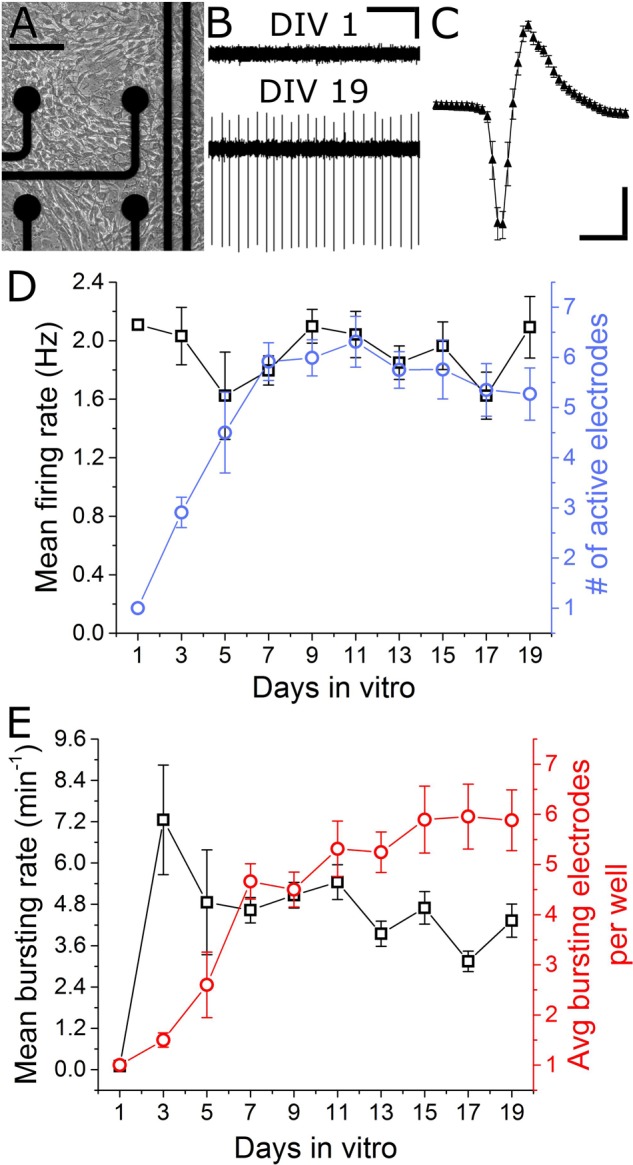
Emergence of spontaneous VH network activity. **(A)** Adhesion and physical network formation on MEA substrates (DIV 4, scale bar indicates 100 μm). **(B)** Traces of continuous filtered data on DIV 1 and DIV 19, illustrating the emergence of well-resolved, high-SNR signals (horizontal/vertical scale bar indicates 1 s/10 μV). **(C)** Average and SD trace of single characteristic unit consistent with time-scale and amplitude of extracellular action potentials (horizontal/vertical scale bar indicates 1 ms/10 μV). **(D)** Mean firing rate and number of active electrodes per well over 19 days *in vitro*. **(E)** Mean bursting rate and average number of bursting electrodes per well over 19 days *in vitro*. Error bars represent SEM.

In total, seven separate VH cultures were derived from seven timed-pregnant female mice for this study. As shown in **Figures 2A,B**, VH cultures on MEAs formed dense physical networks and became spontaneously active. Collections of spike waveforms (or single units) were entirely consistent with the form and time course expected for extracellular action potentials, as shown in **Figure 2C** (Henze et al., 2000). Importantly, the occurrence of multiple units being recorded by a single electrode was rare (<2%). Extracellular recordings were performed at regular intervals (every alternate day from DIV 1) to characterize the emergence and stability of spontaneous spike activity. Spontaneous extracellular action potentials were first observed on DIV 1, when the spike rate was 2.1 Hz (*n* = 1 microelectrodes). Surprisingly, the mean firing rate remained consistent through DIV 19 while the average number of active electrodes per well rose to 6.3 ± 0.5 (**Figure 2D**) by DIV 11. At this time, the cultured VH exhibited an average peak-to-peak spike amplitude of 35.6 ± 2.7 μV, and an overall spike rate of 2.0 ± 0.16 Hz. The signal-to-noise ratio (SNR) for a unit was defined as the mean peak-to-peak spike amplitude over the root mean square (RMS) value of the noise for the corresponding unit. The overall SNR for the baseline recordings was 13.0 ± 1.0, suggesting the detection of well-resolved units from the cultured MNs.

Two types of activity were observed over the culture duration: tonically firing, largely asynchronous activity, and bursting activity, which became increasingly synchronous across electrodes over time. For quantification of network synchrony, we calculated the average area under the normalized cross-correlogram as well as the synchrony index (Luhmann et al., 2016) for each well over time. These two metrics increased from 0.005 ± 0.005 and 0.02 ± 0.01 on DIV 5 to 0.03 ± 0.04 and 0.12 ± 0.11 on DIV 19; both statistically significant increases. Spontaneous bursting activity was observed on single electrodes beginning DIV 3, with a mean burst rate of 7.3 ± 1.6 min^-1^ measured on 1.5 ± 0.14 electrodes/well. No significant changes in mean bursting rate occurred following DIV 7, yet the number of bursting electrodes per well increased to 5.9 ± 0.7 by DIV 15 (**Figure 2E**). Network level bursting, however, peaked on DIV 7 (6.0 ± 1.3 min^-1^) and significantly decreased to 1.8 ± 0.4 min^-1^ by DIV 19. In total, these data suggest a period of functional network maturation over at least 7 DIV, after which the mean firing rate and bursting rate reaches a stable baseline which persists until at least DIV 19.

### Pharmacological Agent Sensitivity

**FIGURE 3:**
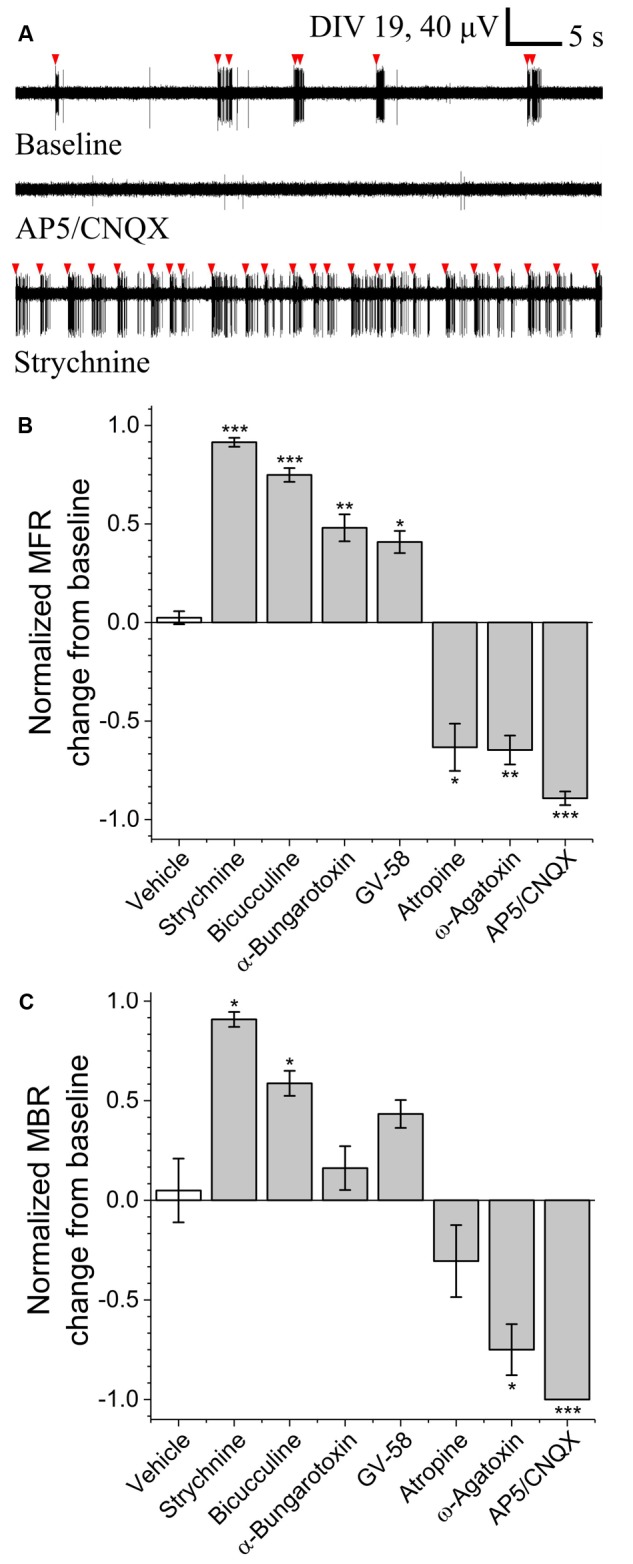
Pharmacological sensitivity of VH networks on MEAs. **(A)** Characteristic continuous filtered traces of baseline activity (DIV 19) and in the presence of AP5/CNQX or strychnine. Bursts indicated by red arrows. DoS normalized mean firing rates **(B)** and mean burst rates **(C)** for various pharmacological treatments and vehicle. ^∗^*p* < 0.05, ^∗∗^*p* < 0.01, ^∗∗∗^*p* < 0.001.

To determine the sensitivity of this assay to pharmacological manipulations, MN network activity measurements were carried out before and after the addition of various pharmacological compounds on DIV 18–19 (**Figure 3A**). In each case, baseline activity was recorded for 10 min, treatments were added to all treated wells simultaneously and activity was recorded for an additional 30 min. No significant onset latency or time-dependent changes in activity were observed over this 30 min interval. Here, we tested sensitivity to agonists and/or antagonists of pre-/post-synaptic receptors involved in MN communication (acetylcholine, glutamate, glycine, and GABA) as well as agonists and antagonists of pre-synaptic P/Q-type calcium channels (GV-58 and ω-agatoxin). **Figures 3B,C** summarizes difference-over-sum (DoS) alterations from baseline for each pharmacological treatment.

Acetylcholine is the principle neurotransmitter released by spinal MNs, responsible for triggering skeletal muscle contractions *in vivo* via the neuromuscular junction. Here, we tested nicotinic and muscarinic acetylcholine receptor (AChR) antagonists α-bungarotoxin (3 μM) and atropine sulfate (10 μM). While atropine sulfate was found to significantly reduce mean firing rate (-0.63 ± 0.12) as compared to baseline, α-bungarotoxin was found to significantly increase mean firing rate (+0.48 ± 0.07). This suggests feed-forward inhibition due to the relatively high density and subunit variety of post-synaptic nicotinic AChRs expressed by inhibitory interneurons (Renshaw cells) versus MNs (Dourado et al., 2002).

To determine whether this assay is sensitive to alterations of glutamate signaling, we tested two glutamate receptor antagonists in combination; *N*-methyl-D-aspartic acid (NMDA) receptor antagonist (2R)-amino-5-phosphonovaleric acid (AP5, 50 μM) and AMPA/kainite receptor antagonist 6-cyano-7-nitroquinoxaline-2,3-dione (CNQX, 20 μM). Treatment with AP5/CNQX caused significant reductions in mean firing rates and mean bursting rates. These data suggest a high degree of sensitivity to glutamate receptor modulation.

To determine whether this assay is sensitive to alterations of inhibitory neurotransmission, we measured activity before and after the addition of glycinergic receptor antagonist strychnine (10 μM) and GABA_A_ receptor antagonist bicuculline (20 μM). Treatment with either strychnine or bicuculline caused significant increase in both mean firing rate (+0.91 ± 0.02 and +0.75 ± 0.04) and mean bursting rates (+0.91 ± 0.07 and +0.59 ± 0.15), indicating a high degree of sensitivity to modulation of both glycinergic and GABAergic receptors.

To determine whether this assay is sensitive to modulation of pre-synaptic P/Q-type calcium channels, we quantified mean firing and bursting rates before and after the addition of GV-58 or ω-agatoxin. Treatment with GV-58 caused a significant increase in mean firing rate (+0.41 ± 0.06), but no significant increase in mean burst rate. Treatment with ω-agatoxin, however, caused significant decrease in both mean firing rate (-0.65 ± 0.07) and mean bursting rate (-0.75 ± 0.26).

### Botulinum Neurotoxin Serotype A – Inhibition, Persistence, and Recovery

**FIGURE 4:**
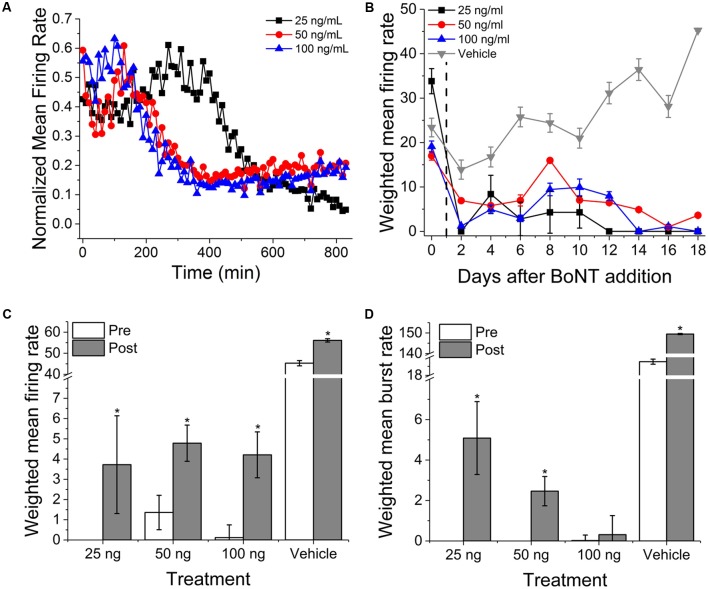
Onset and persistence of VH network inhibition due to BoNT/A intoxication. **(A)** Normalized mean firing rates over 14 h (840 min, 10 min bins) following perfusion with 25, 50, and 100 ng/ml BoNT/A. **(B)** Weighted mean firing rates for 18 days following BoNT/A perfusion. BoNT/A removed after 24 h (indicated by black dashed line). Weighted mean firing rates **(C)** and burst rates **(D)** pre- and post-incubation with 20 μM GV-58. ^∗^*p* < 0.05.

To determine acute and chronic MN sensitivity to BoNT/A using MEAs, we incubated 13-day-old cultures with three BoNT/A concentrations (25, 50, and 100 ng/ml) or vehicle (0.1% DDI water) for 24 h, washed the cultures with fresh SDMEM, and performed repeated measurements of spontaneous network activity over the next 18 days. Acute recordings were also carried out for 14 h immediately following BoNT/A addition to potentially determine the time-dependence of inhibition. Over this 14-h recording phase, all three concentrations of BoNT/A induced between 65 and 89% reduction in normalized mean firing rates (**Figure 4A**). We also observed an initial increase in mean firing rate of approximately 20% prior to network silencing, with the temporal profile of latency/increase/decrease behaving in a concentration-dependent manner. This biphasic profile is consistent with previous studies using motor and cortical neurons (Pancrazio et al., 2014), and may be attributable to increased BoNT/A binding affinity for synaptic vesicle protein SV2A, predominately associated with GABAergic synapses, thereby accelerating the inhibition of GABAergic synapses compared to glutamatergic synapses (Beske et al., 2015).

After 24 h, medium containing BoNT/A was replaced with fresh SDMEM and recordings continued every alternate day for an additional 18 days. Cultures exhibited persistent, significant inhibition of activity over that period of time as compared to baseline and vehicle. By day 18, 25, 50, and 100 ng/ml-treated cultures exhibited 0, 21, and 0% of baseline activity while vehicle-treated mean firing rates had increased to 193% of baseline values. It is important to note, however, that weighted mean firing rates, while dramatically reduced, rarely reached zero for any of the concentrations over the 18-day period (**Figure 4B**). We observed that a very small population of channels continued to fire even after synaptic inhibition, and that this activity was tonic and asynchronous. This is consistent with previous studies of botulinum toxin effects using networks of embryonic stem cell derived neurons (Jenkinson et al., 2017). Following day 18 recordings, cultures were treated with 20 μM GV-58, an N-type as well as P/Q-type calcium channels agonist shown to enhance neurotransmitter release (Tarr et al., 2013). Following GV-58 treatment, weighted mean firing rates increased from 0 ± 0, 1.4 ± 0.9, 0.2 ± 0.6 to 3.7 ± 2.4, 4.8 ± 0.9, and 4.2 ± 1.1 for 25, 50, and 100 ng/ml BoNT/A intoxicated wells, respectively (**Figure 4C**); all statistically significant increases, while vehicle-treated wells increased from 45.3 ± 1.2 to 56.1 ± 0.8. Weighted mean burst rates were also found to increase in the case of 25 and 50 ng/ml, but not 100 ng/ml-treated cultures (**Figure 4D**). These data suggest that our assay can be used to monitor the persistence of intoxication as well as screen for potential recovery agents over time-courses consistent with those of BoNT/A intoxication and recovery (≥15 days).

### Evoked Activity

**FIGURE 5:**
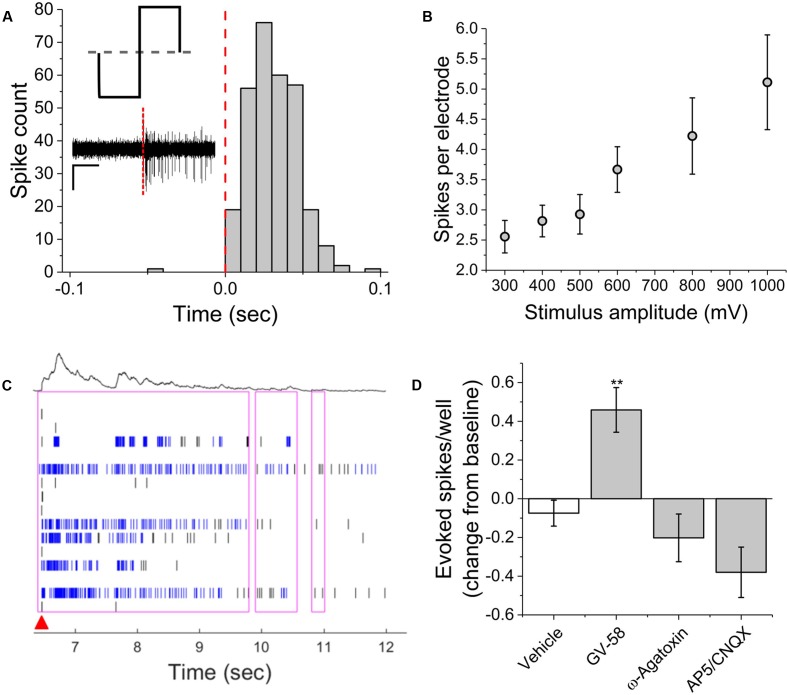
Single-channel and network activity evoked by electrical stimulation. **(A)** Summed peri-event histogram for five consecutive pulses of cathodic-leading charge balanced stimuli (1000 mV at 100 μs per phase). Inset illustrates charge-balanced wave stimulus waveform and shows characteristic filtered continuous trace immediately prior to and following stimulus (red dashed line). **(B)** Amplitude dependence of evoked spikes recorded on stimulating electrode. **(C)** Characteristic raster plot illustrating network evoked activity due to single stimulating electrode. Red arrow indicates time-stamp of stimulation. **(D)** Evoked spikes per well in the presence of pharmacological agents. ^∗∗^*p* < 0.01.

Electrical stimulation using substrate integrated MEAs can be used to ‘pace’ spike or burst events, potentially increasing assay reliability and sensitivity by reducing well-to-well variability. To determine whether this multi-well MEA platform could be used to electrically stimulate MN networks and increase experimental effect size, we conducted exploratory studies in the presence and absence of pharmacological agents. Electric field stimulation of cultured MN networks was applied using recording electrodes on the Axion Maestro multi-well plate system. Single electrodes were randomly selected for stimulation within 28 wells, one electrode per well. We quantified single-channel as well as network-level activity in response to cathodic-leading biphasic square pulses (**Figure 5A**, inset) of varying voltage amplitudes (300–1000 mV) and durations (100–250 μs) at a frequency of 0.1 Hz. **Figure 5A** shows a representative peri-event histogram produced from summing evoked spikes from five consecutive pulses at 1000 mV and 100 μs per phase. We observed an amplitude-dependent increase in the number of spikes per stimulating electrode at 100 μs per phase (**Figure 5B**). Additionally, we observed evoked network-level activity (**Figure 5C**) which increased from 5.3 ± 0.6 to 14.7 ± 2.9 evoked spikes per well at 300 and 1000 mV, respectively.

In the presence of GV-58, normalized well-wide evoked activity increased to 0.46 ± 0.12 of baseline values (*p* < 0.01) (**Figure 5D**). However, there was no significant decrease in evoked spikes/well following the addition either ω-agatoxin or AP5/CNQX. It appeared that in these cases, the pharmacological effects on electrically evoked activity were less prominent than the effects of the same compounds on spontaneous activity. It is possible that electrical stimulation may activate a broader set of ion channels beyond those that typically participate in spontaneous activity. The fact that burst-like activity can be evoked in the presence of the above compounds suggests that sufficient residual synaptic activity may be triggered to produce responses that do not differ significantly from control levels or that the stimulation parameters used here were sufficient to cause direct electric field activation of multiple neurons. However, in the presence of BoNT/A, electrical stimulation failed to evoke network bursts, indicating that synaptic transmission must be at least partially intact for evoked network responses. The parameter space for electrical stimulation is large and the resulting activity is expected to depend on amplitude, phase duration, and interpulse interval. Consequently, further study of the electrical stimulation of pharmacologically treated VH networks will be necessary.

## Discussion

The objectives of the present study were to characterize the spontaneous and evoked activity of dissociated mouse VH neurons cultured on MEAs and to determine the suitability of the multi-well MEA platform for performing acute and long-term pharmacological screening. Our results support five main conclusions: (1) our dissection and culture protocols yield a majority of ChAT-positive neurons (MNs) within a heterogeneous culture of neuronal as well as non-neuronal cells. (2) VH neurons form spontaneously active functional networks which exhibit single unit and bursting activity consistent with the presence of excitatory as well as inhibitory inputs (Darbon et al., 2002). (3) VH networks respond to pharmacological treatments in a manner largely consistent with previous studies using cultured neurons or acute tissue preparations. (4) Multi-well MEAs are able to detect acute and chronic dose-dependent inhibition of network activity due to BoNT/A and partial recovery in the presence of GV-58, supporting previous work performed using acute *ex vivo* tissue preparations as well as *in vivo* rat models of Lambert Eaton Syndrome (Tarr et al., 2013). (5) Single neuron and network activity can be reproducibly evoked in an amplitude-dependent manner using substrate integrated extracellular electrodes.

### Comparison to Previous Work

Gross et al. (1982) first reported spontaneous activity recordings from dissociated mouse spinal neuron cultures using photoetched MEAs in 1982. These cultures were derived from whole spinal cord and served as proof-of-concept for culturing and recording CNS neurons on MEA substrates. More recent studies have also used cultures derived from whole spinal cord to study periodic oscillations in murine spinal cord networks (Keefer et al., 2001; Weiss et al., 2004), botulinum toxin suppression of CNS network activity *in vitro* (Pancrazio et al., 2014), or VH preparations to study pharmacology of VH networks (Zhang and Robinson, 2009). The results reported here largely agree with previous studies of MN activity, pharmacology, and botulinum intoxication while potentially clarifying previously confounding results regarding AChRs. For example, Weiss et al. (2004) reported that exposure to muscarinic AChR (mAChR) antagonist atropine resulted in ‘slight decrease in activity’ while nicotinic AChR (nAChR) antagonist curare resulted in a ‘slight increase in activity.’ Zhang and Robinson (2009), however, reported that while bath applications of acetylcholine significantly increased spike frequency, a combination of n- and m-AChR antagonists (mecamylamine and atropine) did not result in significant changes in overall spike frequency. Here, we report that nAChR antagonist α-bungarotoxin led to significantly increased mean firing rate while mAChR antagonist atropine sulfate led to significantly decreased mean firing rates. In total, these findings suggest that nAChR antagonists may result in feed-forward inhibition *in vitro* due to the relatively high density and subunit variety of post-synaptic nAChRs expressed by inhibitory interneurons (Renshaw cells) versus MNs (Dourado et al., 2002).

### Influence of Culture Heterogeneity

Phenotypically, we observed at least two distinct patterns of neuronal activity: tonic spiking and bursting. Tonically spiking neurons were observed on DIV 1, and their activity persisted beyond DIV 19, even after the addition of neurotransmission inhibitors such as AP5/CNQX and BoNT/A. This suggests that a sub-population within the VH culture does not require excitatory synaptic input in order to fire action potentials. This is consistent with previous studies of VH cultures (Zhang and Robinson, 2009). However, heterogeneity potentially confounds pharmacological screening if the intent is to specifically measure effects on spinal MN excitability; an issue exemplified by our results relating to AChR antagonists. For this reason, several recent studies have made use of highly purified MN preparations for pharmacology and studies of disease-state excitability (Ullian et al., 2004; Barthélémy-Requin et al., 2006; Beaudet et al., 2015; Sances et al., 2016). However, interneurons within the spinal cord provide important, recurrent inhibitory feedback to the MNs and some studies suggest that Renshaw cells have an integral role in movement, though their role is yet to be fully elucidated (Alvarez and Fyffe, 2007; Enjin et al., 2017). Additionally, our culture was largely comprised of non-neuronal support cells (likely a combination of astrocytes and Schwann cells). Non-neuronal support cells have been shown to promote functional synapse formation as well as spontaneous network activity, while highly purified primary spinal MN cultures have exhibited significantly reduced synaptogenesis and multi-fold reduction in spontaneous activity (Ullian et al., 2004). Therefore, many applications may benefit from culture heterogeneity. This requires special consideration in the context of MNs derived from embryonic or induced pluripotent stem cells as it requires the sub-culture and addition of immortalized or primary support cells from animal or human sources. In the case of hiPSC-derived support cells, this process is confounded by the necessary, but cell-type specific (and often proprietary) growth and differentiation supplements necessary for prolonged viability of either cell type *in vitro*.

### Relevance to Embryonic Development

The effects of pharmacological treatments (strychnine, bicuculline, and AP5/CNQX) suggest the presence of excitatory as well as inhibitory neurons within our culture. Moreover, the abolishment of bursting activity in the presence of AP5/CNQX suggests a significant role of glutamatergic neurons in maintaining patterns of VH network bursting *in vitro*. Embryonic spinal cord development also features spontaneous synaptically driven activity as early as E12.5 in mice. This early activity is thought to be largely mediated by GABARs (Scain et al., 2010) but later driven by glutamatergic neurons (O’Donovan, 1999; Law et al., 2014). During this development, spontaneous activity progressively takes the form of coordinated bursting in motor zones, sufficient to drive spontaneous simple movements (twitches, etc.), even following deafferentation or decerebration (Kreider and Blumberg, 2000; Inácio et al., 2016). These spontaneous movements have been implicated in a number of developmental processes, including the formation of sensorimotor circuits necessary for reflex arcs as well as the formation and maintenance of neuromuscular junctions (Sanes and Lichtman, 1999). Pharmacological experiments evaluating age-related aspects of VH network formation may reveal applicability of this model to the study of spinal cord or motor zone development.

### Implications for Neuromuscular Junction Co-culture Assays

Neuromuscular junctions are specialized chemical synapses which serve as the site of action for several neurological disorders and paralytic toxins. Therefore, there has been considerable interest in developing neuromuscular junction models *in vitro*. Several models have relied on open architecture, common-culture of MNs and skeletal myocytes (Das et al., 2007; Drexler et al., 2011; Eckle et al., 2016), while more recent studies have demonstrated the feasibility of compartmentalized MN/myotube co-cultures (Zahavi et al., 2015; Uzel et al., 2016). These preparations exhibit immunohistochemical markers as well as electrophysiological and/or contractile events characteristic of functional neuromuscular transmission. However, this work has sought to quantify alterations of myofiber contraction using cumbersome force transducers (Guo et al., 2011; Smith et al., 2013), regional phase-imaging changes (Zahavi et al., 2015; Vilmont et al., 2016), or excitation–contraction coupling via low-throughput patch-clamp electrophysiology (Ullian et al., 2004; Das et al., 2007; Uzel et al., 2016). Here, we have demonstrated long-term, moderate throughput recordings of phenotypic activity on a platform which does not intrinsically exclude the integration of micro-channel structures for cell-specific compartmentalization. By combining microchannel structures with MEAs, it may be possible to provide high content extracellular action potential recordings from both pre-synaptic MNs and post-synaptic myotubes. Furthermore, we have demonstrated temporally precise and reproducible network-level stimulation, which could serve to drive and temporally correlate pre-/post-synaptic activity.

## Conclusion

For the first time, we have quantitatively characterized the emergence of spontaneous single-unit as well as network activity in a VH culture preparation using MEAs, and that network activity may be evoked by electrical stimulation. Additionally, we have characterized the pharmacological sensitivity of VH cultures to a host of pharmacological agents using a moderate throughput, multi-well MEA system. In the case of BoNT/A, we have further demonstrated that VH cultures on MEAs may be used for moderate throughput, long-term studies of network intoxication and recovery. These findings suggest that VH cultures on multi-well MEAs may be useful as a drug screening or discovery platform.

## Ethics Statement

This study was carried out in accordance with the recommendations of the University of Texas at Dallas’ Institutional Animal Care and Use Committee (IACUC). The protocol was approved by the UTD IACUC. Euthanasia was conducted in accordance with AVMA Guidelines for the Euthanasia of Animals.

## Author Contributions

Conceived and designed the experiments: JP and BB. Performed experiments and data analysis: RA and BB. Wrote the manuscript: BB and JP. All authors contributed to article revision.

## Conflict of Interest Statement

The authors declare that the research was conducted in the absence of any commercial or financial relationships that could be construed as a potential conflict of interest.
